# Protein Stability and Functional Characterization of Intra-Melanosomal Domain of Human Recombinant Tyrosinase-Related Protein 1

**DOI:** 10.3390/ijms21010331

**Published:** 2020-01-03

**Authors:** Monika B. Dolinska, Kenneth L. Young, Claudia Kassouf, Emilios K. Dimitriadis, Paul T. Wingfield, Yuri V. Sergeev

**Affiliations:** 1 National Eye Institute, National Institutes of Health, 31 Center Drive MSC 2510, Bethesda, MD 20892, USA; dolinskam@nei.nih.gov (M.B.D.); kassouf.claudia@gmail.com (C.K.); 2 National Institute of Biomedical Imaging and Bioengineering, National Institutes of Health, 9000 Rockville Pike, Building 13, Room 3N18 Bethesda, MD 20892, USA; dimitre@mail.nih.gov; 3 National Institute of Arthritis and Musculoskeletal and Skin Diseases, National Institutes of Health, 31 Center Dr. - MSC 2350, Bethesda, MD 20892, USA; wingfiep@mail.nih.gov

**Keywords:** tyrosinase-related protein 1, tyrosinase, protein stability, melanin

## Abstract

Pigmentation is the result of a complex process by which the biopolymer melanin is synthesized and packed into melanosomes of melanocytes. Various types of oculocutaneous albinism (OCA), a series of autosomal recessive disorders, are associated with reduced pigmentation in the skin, eyes, and hair due to genetic mutations of proteins involved in melanogenesis. Human tyrosinase (Tyr) and tyrosinase-related protein 1 (Tyrp1) drives the enzymatic process of pigment bio-polymerization. However, within the melanogenic pathway, Tyrp1 has catalytic functions not clearly defined and distinct from Tyr. Here, we characterize the biochemical and biophysical properties of recombinant human Tyrp1. For this purpose, we purified and analyzed the intra-melanosomal domain (Tyrp1tr) for protein stability and enzymatic function in conditions mimicking the environment within melanosomes and the endoplasmic reticulum. The study suggests that Tyrp1tr is a monomeric molecule at ambient temperatures and below (<25 °C). At higher temperatures, >31 °C, higher protein aggregates form with a concurrent decrease of monomers in solution. Also, Tyrp1tr diphenol oxidase activity at pH 5.5 rises as both the pre-incubation temperature and the higher molecular weight protein aggregates formation increases. The enhanced protein activity is consistent with the volume exclusion change caused by protein aggregates.

## 1. Introduction

It has long been known and well documented that pigment production within mammalian melanocytes is largely driven by the catalytic activity of tyrosinase (Tyr), a binuclear multifunctional enzyme whose primary role centers on the initial and rate-limiting reactions in melanin biosynthesis. Although Tyr’s catalytic function tends to be the focus of melanogenesis, there are two other tyrosinase-related proteins, Tyrp1 and Tyrp2, which have roles that have yet to be clearly defined in humans. The crystal structure of Tyrp1 is currently available [1]. All human tyrosinases share a remarkable number of characteristics including melanocyte-specific expression, transmembrane motifs, localization to the melanosome, an EGF protein interaction motif, similar N-glycosylation sites, and similar protein sequences of catalytic domain. In the melanosome membrane, all three proteins may form a hetero-oligomeric complex, which can play a role in the stabilization of Tyr and regulation of its enzymatic activity [2,3,4]. 

Previously, the full-length of human recombinant Tyr and its intra-melanosomal domain (residues 19–469) were purified and biochemically characterized [5,6]. We have shown that the domain is a soluble monomeric glycoprotein with both monophenolase and diphenol oxidase enzymatic activities. Also, a full-length Tyr, which is a Type 1 membrane protein, when solubilized in detergent micelles, exists as an enzymatically active monomer. The binding sites for glycosylation, which is critical for protein stability, obtained for human Tyr from larval expression are localized similar to the positions from human species [5,7]. In addition, we suggested a direct link between OCA1 mutations, conformational stability, and enzymatic activity of Tyr intra-melanosomal domains [8].

Mutations in Tyr and Tyrp1 result in oculocutaneous albinism (OCA1 and OCA3, respectively), a heterogeneous group of congenital developmental pigmentation disorders [9]. In contrast to mutations in Tyr, mutations in Tyrp1 affect the quality of melanin synthesized rather than the quantity. To date, 36 mutations (including 17 missense) in *TYRP1* gene associated with OCA3 were detected (http://www.hgmd.cf.ac.uk/ac/index.php). OCA3 (MIM 203290) is a rare disease that affects 1 in 1,000,000 individuals in the world population (1 in 8500 in Africa) [10,11,12]. Affected individuals usually present one of two phenotypes: rufous OCA (ROCA), characterized by red-bronze skin, blue or brown irises, and ginger-red hair; or brown OCA (BOCA), characterized by light to brown or tan skin and light to brown hair. In both cases, skin pigmentation can increase with age. Foveal hypoplasia, strabismus, nystagmus, and photophobia are common visual function abnormalities for most types of albinism but are not always present in OCA3. 

The human Tyrp1 is a Type 1 membrane-bound protein with an alpha helix spanning the membrane of the melanosome. Tyrp1 is a glycoenzyme containing at least six *N*-glycosylation sites that help maintain both the stability and function of the protein. It has two bound divalent metals (zinc or copper), coordinated by six histidine residues, that remain essential for catalytic function [13,14]. Although its exact functions remain unclear, Tyrp1 is a melanocyte-specific gene product involved in the downstream biochemical events in the subsequent modulation of melanin formation [15]. Moreover, Tyrp1, the most abundant protein of the melanosome, is involved in maintenance of melanosome structure, can affect melanocyte proliferation as well as cell death, and may regulate or influence the type of melanin synthesized [2,16,17,18,19]. Two types of melanin are produced by melanocytes: black-brown eumelanin, which is responsible for black or brown hair and dark skin; and red-yellow pheomelanin, predominantly seen in individuals with red hair and freckles [20]. While Tyr is involved in both eumelanin and pheomelanin synthesis, Tyrp1 is only involved in eumelanin synthesis. Thus, loss of Tyrp1 oxidase activity does not lead to a complete loss of pigment, but only a change in the amount and biochemical character of eumelanin or the eumelanin/pheomelanin ratio [19].

Tyrp1 is synthesized in the endoplasmic reticulum (ER), where it undergoes posttranslational modifications and protein folding [21]. Tyrp1 then passes through the Golgi, where maturation ensues and it is subsequently transported to its final destination in melanosomes, where it might stay in the complex with Tyr and Tyrp2 and plays a role in melanin biosynthesis [4]. However, its precise function is still unspecified and shown to be different between humans and mice. Mouse Tyrp1, much like Tyr, shows tyrosine monophenolase activity, but lacks DOPA oxidase activity [22]. Additionally, Tyrp1 catalyzes the oxidation of 5,6-dihydroxyindole-2-carboxylic acid (DHICA) into indole-5,6-quinone-2-carboxylic acid (IQCA), though its DHICA oxidase activity is controversial. In the presence of 3-Methyl-2-benzothiazolinone hydrazine hydrochloride (MBTH) DHICA is trapped in a stable colored adduct with the hydrazine group, ending the reaction [23]. Hearing’s group showed that human Tyrp1 does not possess DHICA oxidase activity in contrast to murine Tyrp1 [24]. Tyrp1 has also been attributed with other catalytic functions such as dihydroxyindole (DHI) oxidase or dopachrome tautomerase [18,25]. Unfortunately, previous Tyrp1 studies fail to provide enough information about the protein function, possible substrates, ligands, and inhibitors compared to the abundant biochemical information presented in the BRENDA database on tyrosinases from different species (https://www.brenda-enzymes.org).

Recently, we have shown that the incubation of human intra-melanosomal domain of Tyr with excessive amounts of human intra-melanosomal domain of Tyrp1 (Tyrp1tr) increases the stability of Tyr over time. In addition, our data indicated that this mechanism does not appear to involve the formation of stable hetero-oligomeric complexes of Tyr and Tyrp1 to maintain the protective function [14]. Here, we analyzed how the biochemical and biophysical properties of Tyrp1tr and its interactions behave under physiological temperature, since all our purification steps and association experiments were done at ambient temperature. For this purpose, the thermal retention (4–43 °C) of Tyrp1tr was analyzed. For some experiments we chose 4, 25, 43 °C (as storage, experimental, and heat shock temperatures, respectively), as well as 31 °C, as the highest activity temperature for thermal-sensitive Tyr mutants [5]. Temperature studies suggested that Tyrp1tr is a monomeric molecule at 25 °C and below (<25 °C) ambient temperatures. At higher temperatures, >31 °C, higher protein aggregates form with a concurrent decrease of monomers in the solution. In addition, we analyzed Tyrp1tr for protein stability and enzymatic function in conditions mimicking the environment within melanosomes and ER. We have shown that human Tyrp1tr has DHICA oxidase activity; however, calorimetrically seen only in the presence of MBTH. This activity varied at different pH levels and was dependent on the presence of a reducer in buffers. At higher temperatures, the protein activity is increased in contrast to a loss of molecules in a monomeric state, which is consistent with the concurrent volume exclusion change caused by protein aggregates.

## 2. Results

In earlier work, we have shown that Tyrp1tr exists as a monomeric protein with a weight-average molecular weight of 59.2 ± 2.3 kDa, assuming 10% carbohydrates mass due to protein glycosylation [14]. Here, using pure Tyrp1tr (Figure S1), we confirmed the monomeric state of the protein at ambient temperature.

### 2.1. Sedimentation Velocity 

**Figure 1:**
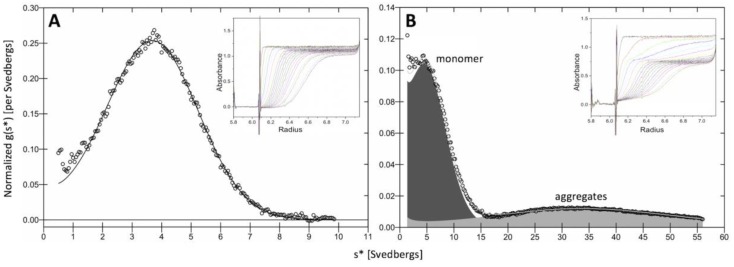
Sedimentation velocity analysis of Tyrp1tr sample. Graphs show the sedimentation coefficient distributions in the samples stored at 4 °C (Panel **A**) and pre-incubated at 37 °C × 24 h (Panel **B**). Raw data are shown by open circles; a line represents the best fit. The inserts show the SV analysis of experiments conducted at 40,000 rpm, 20 °C.

Sedimentation velocity (SV) experiments indicated that in samples stored at 4 °C and analyzed at 20 °C, >90% of Tyrp1tr was monomeric with an expected molecular weight of 59.5 ± 0.6 kDa and sedimentation coefficient of 3.98 S (± 0.01), (Figure 1A). Only 55% of the total protein remained monomeric if the sample was pre-incubated at 37 °C × 24 h prior to run (Figure 1B). The remaining protein formed higher molecular aggregates, which are shown by a second peak on the right with significantly increased value of sedimentation coefficient (~36 S).

### 2.2. Atomic Force Microscopy

**Figure 2:**
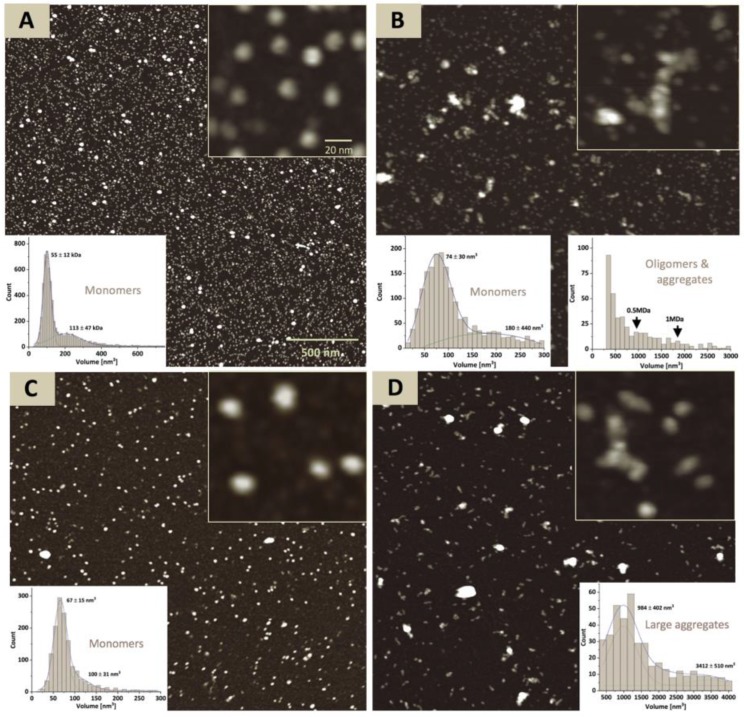
Atomic force microscopy of Tyrp1tr. The full-field images of the Tyrp1tr stored at 4 °C (Panel **A**), pre-incubated at 37 °C × 24 h (Panel **B**), and fractions concentrated from the Peak A (Panel **C**) and Peak B (Panel **D**) (see Figure 3). The inserted graphs show the particle volume distributions for the monomers (Panels **A** and **C**) and monomers and/or larger aggregates (Panels **B** and **D**). The inserted pictures show the enlarged fragments of each image.

For atomic force microscopy (AFM) experiments the Tyrp1tr was stored at 4 °C or pre-incubated at 37 °C × 24 h, then deposited onto an ASP mica surface and imaged in ambient air. Tyrp1tr at the ambient temperature appears monodisperse and shows a uniform distribution of the protein volumes of 94 ± 51 nm^3^, which is consistent with a monomer (Figure 2A). At 37 °C × 24 h condition particle analysis reveals the presence of the 40% monomer agreed with the 55% estimate from sedimentation velocity. Also, about 60% of the protein was found in polydisperse aggregates ranging from small oligomers to large assemblies (Figure 2B).

### 2.3. Gel-Filtration Chromatography

**Figure 3:**
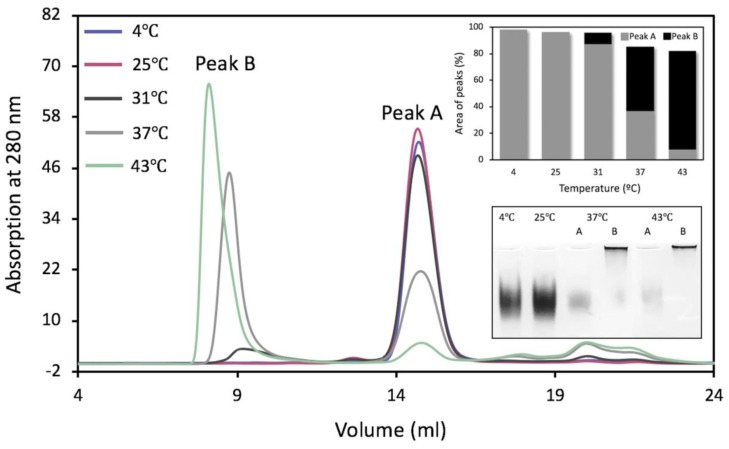
Gel-filtration chromatography of Tyrp1tr. The graph shows the chromatography profiles of Tyrp1tr pre-incubated for 24 h, at 4 (blue line), 25 (pink line), 31 (black line), 37 (gray line), and 43 °C (green line) and eluted from the Superdex 200 Increase GL 10/300 column as Peaks A and/or Peaks B. The top insert shows the % of the peak’s area calculated from the Unicorn 7.0 software (GE Healthcare Bio-Sciences, Pittsburgh, PA, USA), as well as the ratios between Peaks A (gray bars) and Peaks B (black bars). The bottom insert shows the native gel of Tyrp1tr fractions collected from Peaks A and B. From the left: fractions pulled from Peak A of Tyrp1tr pre-incubated for 24 h, at 4 °C (4 °C); fractions pulled from Peak A of Tyrp1tr pre-incubated for 24 h at 25 °C (25 °C); fractions pulled from the Peak A (37 °C, A) and Peak B (37 °C, B) of Tyrp1tr pre-incubated for 24 h at 37 °C; fractions pulled from the Peak A (43 °C, A) and Peak B (43 °C, B) of Tyrp1tr pre-incubated for 24 h, at 43 °C.

To characterize protein stability using gel-filtration chromatography (GF), Tyrp1tr samples were pre-incubated at five different temperatures for one or 24 h: 4, 25, 31, 37, and 43 °C (Table S1). After 24 h of pre-incubation at 4 or 25 °C, Tyrp1tr was eluted from the Superdex 200 Increase 10/300 GL column as single monomeric peaks with an apparent molecular weight of 57.6 ± 1.9 and 59.6 ± 1.0 kDa, respectively (Peak A, Figure 3, Table 1). These results were consistent with 54.95 kDa previously reported [14].

However, the 24 h pre-incubation at higher temperatures (>31 °C) showed an additional peak at the higher molecular weight (Peak B), which was close to the exclusion limit for the column, 1300 kDa (Figure 3). The relative fraction of Peak B rises as temperature increases. Indeed, the formation of Peak B exhibited an increase of 9.0% at 31 °C, 56.9% at 37 °C, and 90.7% at 43 °C (Figure 3, top insert). Predictably, the period of pre-incubation affects Peak B formation. The Peak B construct increased from 17.7% to 58.9% when the sample was pre-incubated at 37 °C for 1 h and 24 h, respectively (Figure S2). After pre-incubation at 43 °C for 1 h or 24 h, Peak B increased from 60.2% to 90.7%, respectively (Figure S3). It is also important to point out that fractions collected from any Peak B showed no DHICA activity. Fractions from the monomeric peaks (Peak A) corresponding to the single bands migrated well at the same time on the native gel electrophoresis. Conversely, the migration of the fractions from Peak B, which moved towards the higher molecular weight, was markedly stunted, with most of the protein retained at the loading position of the gels (Figure 3, bottom insert).

Moreover, the analysis of Peaks A and B eluted from the Superdex 200 Increase 10/300 GL column from the sample incubated at 37 °C was in congruence with results of AFM (Figure 2C,D). In AFM, a sample from Peak A showed a relatively uniform population of monomers with a volume of 67 ± 15 nm^3^, whereas the sample from Peak B revealed a population of large aggregates with a volume range of 948 ± 402 nm^3^–3412 ± 510 nm^3^.

### 2.4. Dynamic Light Scattering

**Figure 4:**
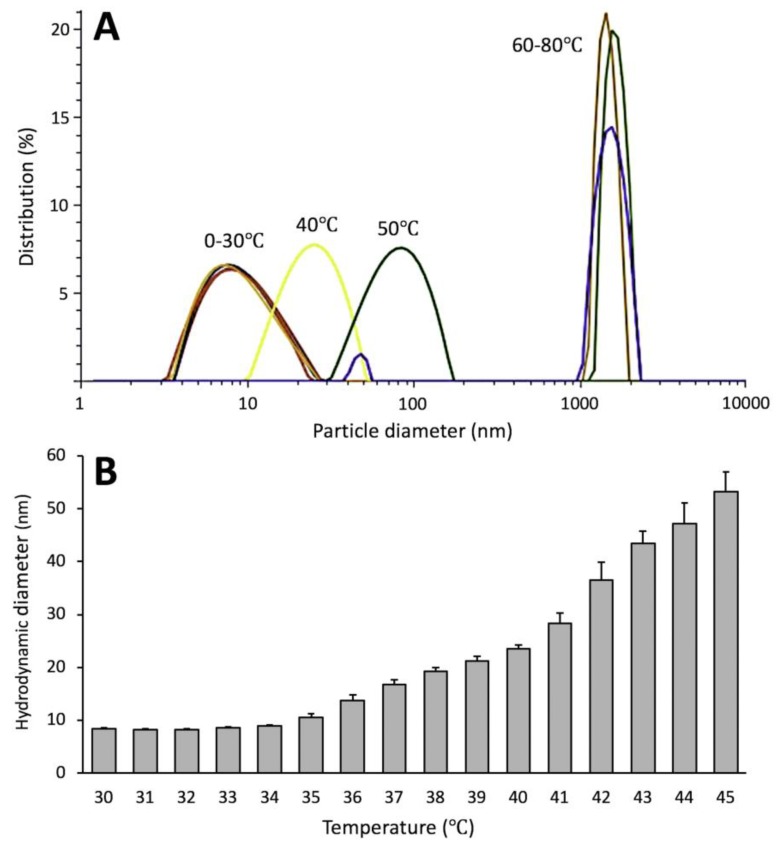
Thermostability of Tyrp1tr determined by DLS. Panel **A**: Particle diameter distribution by intensity for Tyrp1tr at 2 mg/mL measured at temperature range of 0–80 °C, at 10 °C intervals. Panel **B**: Hydrodynamic diameter of the same Tyrp1tr protein sample measured at the temperature range of 30–45 °C, at 1 °C intervals.

The proteins pooled separately from Peaks A and B were subjected to dynamic light scattering (DLS) measurements to determine the molecular sizes of proteins from the peaks (Table 1). The hydrodynamic diameter (D_h_) was measured for both peaks increased with temperature. However, D_h_ indicate only the apparent size of the hydrated particle, which could be different from the size determined from the crystallographic model; it is still a convenient way to estimate a relative changes in Tyrp1tr structure at different temperature. However, D_h_ of Peak B for protein pre-incubated at 37 or 43 °C clearly showed a correspondent increase of D_h_ to 52.8 ± 0.4 and 71.5 ± 1.3 nm, suggesting possible protein aggregation. In another DLS experiment, Tyrp1tr was measured during gradual temperature increases from 0–34 °C and showed D_h_ values changing around 8 nm, which is consistent with a monomer (Figure 4, Table S2). However, above 34 °C, the D_h_ of Tyrp1tr started to gradually increase with rises in temperature suggesting a formation of higher molecular weight protein species (Figure 4, Table S2).

### 2.5. Diphenol Oxidase Activity of Tyrp1tr

**Figure 5:**
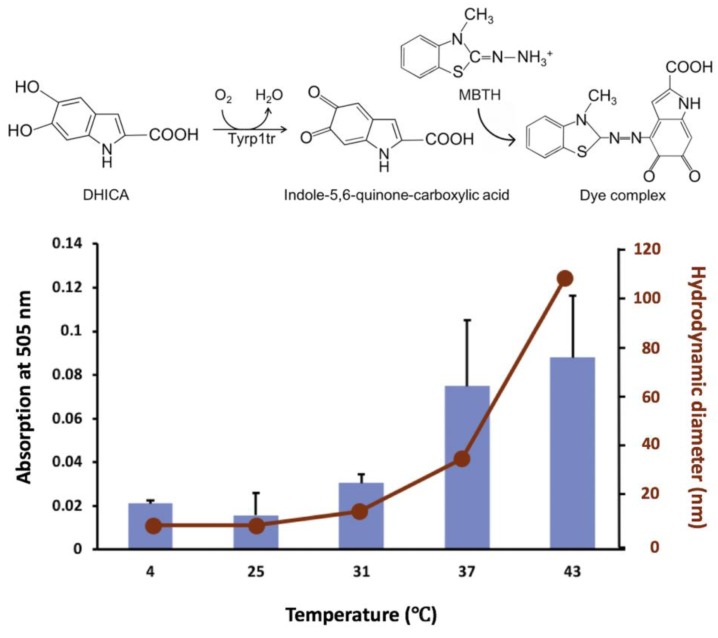
A relationship between the diphenol oxidase activities and hydrodynamic diameters of Tyrp1tr_._ Tyrp1tr diphenol oxidase activities (blue bars) were measured in the presence of DHICA and MBTH. The samples were pre-incubated in GF buffer (see Material and Methods), pH 7.2, for 180 min at 4, 25, 31, 37, and 43 °C. The reaction mixture containing 3 mM DHICA and 3 mM MBTH was monitored by measuring the IQCA/MBTH formation (see reaction) at 505 nm. Hydrodynamic diameter (red dots) was measured by DLS in similar conditions.

The formation of dark pigment of the IQCA/MBTH complex was monitored spectrophotometrically at 505 nm (Figure 5). For this purpose, the protein, DHICA, and MBTH were incubated for 180 min at different temperature conditions: 4, 25, 31, 37, and 43 °C. The diphenol oxidize activity of Tyrp1tr increased with temperature as characterized by absorption values of 0.021 ± 0.001, 0.016 ± 0.010, 0.030 ± 0.004, 0.075 ± 0.030, and 0.088 ± 0.028 mOD/min measured at corresponding temperatures of 4, 25, 31, 37, and 43 °C, respectively. However, in DLS experiments, D_h_ showed a monomer at 4 and 25 °C (7.2 ± 0.1 and 7.4 ± 0.2 nm, respectively), then the diameter of the protein increased gradually to show 13.0 ± 0.6, 34.5 ± 0.9, and 108.2 ± 1.7 nm for 31, 37, and 43 °C, respectively (Figure 5).

### 2.6. Tyrp1tr under pH 5.5 Condition

**Figure 6:**
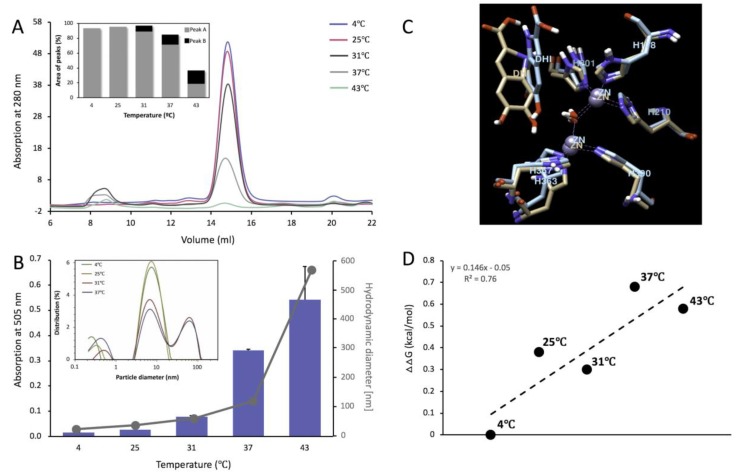
Tyrp1tr analyzed at pH 5.5 mimicking conditions within the melanosome. Panel **A**: Graph shows the chromatography profiles of Tyrp1tr pre-incubated for 24 h, at 4 (blue line), 25 (pink line), 31 (black line), 37 (grey line), and 43 °C (green line) and eluted from the Superdex 200 Increase GL 10/300 column. The insert shows the % of the peak’s area calculated from the Unicorn 7.0 software (GE Healthcare Bio-Sciences, Pittsburgh, PA, USA), as well as the ratios between Peaks A (grey bars) and Peaks B (black bars). Panel **B**: Tyrp1tr diphenol oxidase activities (blue bars) measured using DHICA as a substrate in the presence of MBTH after pre-incubation for 180 min at 4, 25, 31, 37, and 43 °C and monitored by measuring the IQCA/MBTH formation at 505 nm. Hydrodynamic diameter (grey dots) was measured after pre-incubation of Tyrp1tr for 180 min at 4, 25, 31, 37, and 43 °C. The insert shows particle diameter distribution by intensity for Tyrp1tr at 2 mg/mL measured at temperature 4, 25, 31, 37, and 43 °C. Panel **C**: The superposition of Tyrp1 atomic models was obtained by 6 ns molecular dynamics equilibration at 4 °C (beige) and 43 °C (blue). The best molecular poses of DHICA molecules (labeled as DHI) for each model are shown. Only Zn-atoms and histidine residues are presented in each case. Panel **D**: Free energy changes, ΔΔG of Tyrp1 DHICA docking at pH 5.5 and different temperatures. ΔΔG was defined as a difference between Gibbs free energy changes of the DHICA/Tyrp1 complex and free energy changes for individual molecules and presented relative to that of 4 °C measurements.

At pH 5.5 and ambient temperature, the protein elutes from the Superdex 200 Increase 10/300 GL column as a single monomeric peak for 4 and 25 °C with an apparent molecular weight of 54.3 ± 0.9 and 55.0 ± 0.9 kDa, respectively (Figure 6A, Table 1). When Tyrp1tr was incubated at >31 °C x 24 h, at pH 5.5, the sample appeared cloudy and had been clarified by centrifugation at 13,000 rpm for 30 min. The clarified supernatant remained mostly monomeric, as indicated by GF, AFM, and SV (Figure 6A and Figure S4). Under these conditions, most of the Tyrp1tr eluted as several peaks with an apparent molecular weight of 54.0 ± 0.5, 56.9 ± 0.9 and 57.6 ± 1.9 kDa, respectively. The relative ratio of monomer-to-higher MW associate (Peak A/Peak B ratio) decreased as 11.0, 5.7, and 1.0 at increased pre-incubation temperatures of 31, 37, and 43 °C, respectively (Figure 6A, insert). After incubation at 43 °C, more than 80% of protein had aggregated and remained in the pellet.

AFM further confirmed that at pH 5.5 and ambient temperature the protein volume distribution exhibited a major peak at 112 ± 26 nm^3^, consistent with a monomer (Figure S4A). There was also a tail in the higher volume range of the histogram—apparently close to 24% of the total protein might have volumes closer to a dimer. However, the broad distribution of volume around the mean and the quality of the Gaussian fit to the data make such a conclusion doubtful. When exposed to 37 °C, much of the protein (~75%) formed large polydisperse aggregates. Only about 4–5% of the total protein mass remained in monomer form while another 20% formed small (dimers to hexamers) oligomers (Figure S4B).

As we showed by velocity scan, 90% of protein kept at 4 °C remained monomeric with expected molecular weight of 60.8 ± 0.8 kDa and s = 4.1 ± 0.0 (Figure S4C). Sample incubated at 37 °C, pH 5.5, for 24 h and then clarified by centrifugation for 14,000 rpm for 10 min (~40% of total sample) appeared monomeric with molecular weight of 57.3 ± 1.0 kDa and s = 4.1 ± 0.0 (Figure S4D).

The diphenol oxidize activity of Tyrp1tr, at pH 5.5, grows significantly to values of 0.016 ± 0.001, 0.026 ± 0.010, 0.078 ± 0.003, 0.341 ± 0.005, and 0.542 ± 0.132 mOD/min at corresponding temperatures of 4, 25, 31, 37, and 43 °C, respectively (Figure 6B). Values of D_h_ suggest the presence of monomers at 4 °C (8.40 ± 0.02 nm). Higher aggregates with hydrodynamic radii of 20.3 ± 1.4, 45.4 ± 3.3, 105.5 ± 10.4, and 557.1 ± 31.2 nm were observed at 25, 31, 37, and 43 °C, respectively. Thus, Tyrp1tr diphenol oxidize activity at pH 5.5 increases with the rise in pre-incubation temperature and the simultaneous increase of higher aggregate forms characterized by larger D_h_ (Figure 6B, insert).

To explain the temperature properties of Tyrp1tr/DHICA interaction at pH 5.5, computer simulations of temperature-dependent association of DHICA using the atomic model of Tyrp1 were performed. DHICA molecules were docked to the Tyrp1 active site at different temperature conditions (Figure 6C and Figure S5). The binding energies were calculated as 6.0, 6.4, 6.3, 6.7, and 6.6 kcal/mol at corresponding temperatures of 4, 25, 31, 37, and 43 °C, respectively. The changes of binding free energies increase as the temperature increases, as shown in Figure 6D.

### 2.7. Tyrp1tr Catalytic Activities at pH 5.5 and pH 7.2

**Figure 7:**
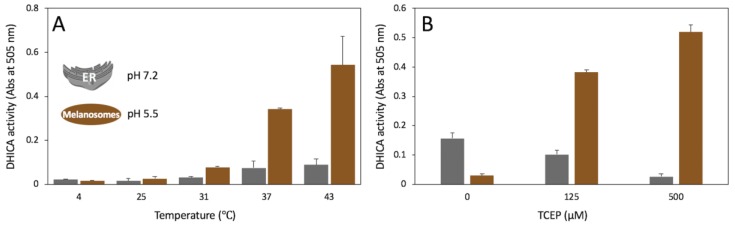
Tyrp1tr diphenol oxidase activity measured to mimic pH conditions in the ER (pH 7.2) and melanosomes (pH 5.5). Panel **A**: Tyrp1tr diphenol oxidase activities measured at pH 7.2 (grey bars) and 5.5 (brown bars) using DHICA as a substrate. The reaction mixture containing 3 mM DHICA and 3 mM MBTH in GF buffer including 50 µM TCEP, pH 7.2 or 5.5, was incubated for 180 min at 4, 25, 31, 37, and 43 °C and monitored by measuring the IQCA/MBTH formation at 505 nm. Panel **B**: Tyrp1tr diphenol oxidase activities measured at pH 7.2 (grey bars) and 5.5 (brown bars) using DHICA as a substrate in the presence of different concentrations of TCEP. The reaction mixtures containing 3 mM DHICA, 3 mM MBTH, and 0, 125, or 500 µM TCEP in GF buffer, pH 7.2 and 5.5 were incubated for 180 min at 37 °C and monitored by measuring the IQCA/MBTH complex formation at 505 nm.

The diphenol oxidase activity of the Tyrp1tr was measured at different pH, temperature, and reducing conditions (Figure 7). Tyrp1tr demonstrated the highest enzymatic activity at 37 and 43 °C, especially at pH 5.5 (Figure 7A). Figure 7B shows the DHICA activity of Tyrp1tr measured at pH 7.2 and 5.5 in different concentrations of a reductant. Under conditions with high concentrations of the reducing agent, TCEP (tris(2-carboxyethyl) phosphine), present in the buffer, the enzymatic activity of the Tyrp1tr was significantly higher at pH 5.5. It is interesting that when no TCEP is present in the buffer, the DHICA activity of Tyrp1tr is ~5 times higher at pH 7.2. When the TCEP concentration is increased, the Tyrp1tr activity is higher at pH 5.5 than at pH 7.2 with 3.7 and 19.5% for 125 and 500 µM TCEP, respectively. Also, the DHICA activity measured at pH 7.2 has a trend to decrease with the increase of the reducer concentration.

## 3. Discussion

Here, we characterized biochemical and biophysical properties of recombinant human tyrosinase-related protein 1. For this purpose, we purified and analyzed the Tyrp1tr for protein stability and enzymatic function in conditions which mimic the environment in melanosomes and the endoplasmic reticulum. Temperature studies suggest that Tyrp1tr is a monomeric molecule at the temperature close to ambient (25 °C) or below. At higher temperatures, >31 °C, higher MW protein aggregates formed concurrently with the decrease of monomers in solution. Moreover, we have shown that human Tyrp1tr has DHICA oxidase activity; however, calorimetrically seen only with the presence of MBTH. This activity varied at different pH and is dependent on the presence of a reducer in buffers. At higher temperatures, the protein activity is increased in contrast to a loss of molecules in a monomeric state, which is consistent with the concurrent volume exclusion change caused by protein aggregation.

Previously we have shown that, in conditions mimicking the ER (pH 7.2) [26] and melanosome (pH 5.5) [27] environments, Tyrtr efficiency in the presence of excess Tyrp1tr is ≈55% greater than the control value at pH 7.2 and 200% greater at pH 5.5, suggesting that the incubation with excess Tyrp1tr protects Tyr by increasing its stability over time, especially in melanosomal like conditions [14]. Thereby, we confirmed in vitro the previous in-vivo–cellular work results, which showed that Tyrp1 can maintain the stability of Tyr and modulate its catalytic activity in eumelanin synthesis [22]. We also illustrated that this mechanism does not appear to involve the formation of stable hetero-oligomeric complexes of Tyr and Tyrp1 to maintain the protective function in contrast to in vivo studies, which suggests a stable hetero-dimeric complex of Tyr and Tyrp1 in murine melanosomes [4,14]. Certainly, we might be missing a cell-specific effect in our in vitro experiments and, therefore, cannot exclude stabilizing transitory interactions between tyrosinases within the melanosome. However, to explore this further we decided to analyze how the proteins and their interactions behave at physiological temperature rather than the normally used room temperature.

At the ambient or physiological temperatures, hydrophobic residues of proteins are located mostly in the interior of the folded structure. As the temperature increases, the chances of the hydrophobic residues being on the surface are increased, eventually leading to protein aggregation [28]. In the case of Tyrp1tr, we showed, when Tyrp1tr was analyzed at the ambient temperature, that most of the protein was monomeric. Moreover, protein conformational changes can establish transient interactions between the molecules leading to aggregation at body temperature (37 °C). However, when the sample was pre-incubated at 37 °C for 24 h, only half of the Tyrp1tr remained monomeric and the rest formed higher molecular aggregates with a sedimentation coefficient of ~36 S (Figure 1 and Figure 2). When the incubated Tyrp1tr was eluted from a Superdex 200 column, an additional peak with higher molecular weight appeared (Figure 3). Also, the area of the monomer peak, decreased up to ~50% and the ratio between monomer/higher assemblies was roughly 1:1. The additional high molecular weight peak, although smaller, also appeared after the incubation of Tyrp1tr at 31 °C. At heat shock temperature (43 °C), the monomers/large assemblies’ ratio dramatically decreased up to 0.1. The average D_h_ of Tyrp1tr determined by DLS at 4 °C was ~8.5 nm and did not change noticeably when temperature was increased up to 34 °C (Figure 4). This number corresponds to the diameter of single-molecule of protein assuming a layer of 30% surrounding water, which was estimated as 7.37 nm from the accessible area or 9.00 nm from the Van-der-Waals surface of the Tyrp1 atomic structure using YASARA STRUCTURE (www.yasara.org).

Above 35 °C the D_h_ of Tyrp1tr gradually increased up to 45 °C, probably as an effect of a less tense state of the protein molecule and, finally, unfolded and aggregated above 50 °C (Table S2). Interestingly, the increase of D_h_ of Tyrp1tr was not reversible at any point. In contrast to pH 7.2, when Tyrp1tr was incubated above 31 °C, at pH 5.5 (melanosomal condition), ~60% of protein aggregated as was evidenced by cloudy-turbid like appearance and had to be clarified by centrifugation. At pH 5.5 (just below Tyrp1tr isoelectric point, ~6.01) the protein was more susceptible to higher temperature, which led to stronger aggregation than at pH 7.2; although in the clarified supernatant mostly monomeric forms of Tyrp1tr were detected (Figure 6A and Figure S4). It is also important to point out that after the incubation at 43 °C more than 80% of protein aggregated and remained in the pellet. At pH 5.5 the protein is monomeric only at 4 °C, at which point it starts to gradually increase in size with increase in temperature. In general, the aforementioned analysis points to a decrease in Tyrp1tr monomers, when the protein was subjected to 24 h incubation at > 31 °C (pH 7.2 and 5.5) that is proportional to increase in temperature. The non-monomeric protein appears as heterogeneous aggregates rather than regular oligomers, which is different from previous in vivo studies in murine melanocytes where regular oligomers were proposed [4].

Tyrp1 is known as an enzyme catalyzing DHICA oxidation [22,23,29]. However, this catalytic activity appears to be species-specific as it has been proven only in the cells of mice [24]. Recently Dijkstra’s group demonstrated DHICA activity of human recombinant Tyrp1 substituted with copper ions instead of zinc [1]. We previously showed using ICP-MS that our human recombinant Tyrp1tr contained both Cu^2+^ and Zn^2+^ ions [14]. Here, we demonstrated the DHICA activity of Tyrp1tr and showed its activity increased with higher temperature, although the protein lost its monomeric state at the same time (Figure 5). Interestingly, we have observed a significant difference of the Tyrp1tr catalytic activity between pH 7.2 and pH 5.5 conditions. At pH 7.2, assumed Tyrp1tr activity increased by 60-fold at 43 °C compared to that of at 4 °C (Figure 5, Table 1). In contrast, the activity of Tyrp1tr at pH 5.5 at the same pre-incubation condition increased ~180-fold compare to that of at 4 °C (Figure 5 and Figure 6B). Such differences in catalytic activity could be explained by using Tyrp1 protein structure and the effect of volume exclusion due to aggregation of the protein. Indeed, molecular dynamics simulations suggest some binding improvements with the temperature increase. Figure 6C shows the superposition of active sites of 2 atomic models of Tyrp1 obtained by equilibration using 6 ns molecular dynamics at 4 °C (grey) and 43 °C (blue), respectively. The best molecular poses of DHICA molecule were obtained using molecular docking as described in Material and Methods paragraph. The increase of catalytic activity at 43 °C associated with the tighter binding of DHICA molecule in the active site is shown in Figure 6C,D. The tighter binding is related to the increased accessibility of an active site at higher temperatures. The discrepancies in Tyrp1 activity at different pHs could be related to the protein aggregation, which was higher at acidic pH. The role of aggregation will be very similar to the effect of macromolecular crowding as we discussed previously [14]. In molecular crowding, the equilibrium constants may be increased by 2 or 3 orders of magnitude, protein activity increases very significantly, and local concentration of substrate will also rise [30].

This increase of catalytic activity was significant at both pH condition, but it was much more pronounced at the acidic pH. This indicates that such an increase could be important at the late phase of melanosomal development when melanin fibers growth is orchestrated with the production of free radicals and decreasing of the free space available for the melanin pathway reactions.

Moreover, the enzymatic activity of Tyrp1tr was significantly higher at pH 5.5 when the reducing agent, TCEP, was present in the buffer (Figure 7A). Without TCEP, the DHICA activity of Tyrp1tr was ~5 times higher at pH 7.2. Tyrp1tr activity at pH 5.5 increased with increasing concentrations of TCEP, in contrast to the activity measured at pH 7.2. These findings could indicate the involvement of sulfhydryl groups of Tryp1tr while in acidic environments. In contrast, the reduction properties of the TCEP bond breaker are optimized for a neutral pH (Thermo Scientific, document 77720). In Figure 7B, the decrease of DHICA activity at pH 7.2 with the reducer increase suggests a protein activity loss due to the reduction of disulfide bridges.

Indeed, the crystal structure of the Tyrp1 protein (RCSB file: 5M8L) is stabilized by 7 disulfide (S–S) bridges. Stability of the structural motif Q25-D123 located at the beginning of protein chain is maintained by 5 S–S bridges: C30–C41, C42–C65, C56–C99, C101–C110, and C113–C122. Two additional S–S bonds stabilize the tyrosinase domain: C261–C258 and C290–C303. Interestingly, the majority of S–S bridges will be broken by strong reducing conditions at pH 5.5 (Figure 7B). At first glance, this does not explain the increase in Tyrp1tr activity in the presence of a reductant. However, the N-terminus motif will be destabilized, unfolded, and prone to protein aggregation under these conditions. The effect of volume exclusion could be caused by growing protein aggregates, which are excluding and decreasing the volume accessible to the ligand. As a result, the local concentration of the ligand will increase and will cause the concurrent increased activity of Tyrp1, which was observed in our study. Such a role of protein aggregation in Tyrp1 activity is consistent with the results of the temperature increase presented in Figure 7A.

On the other hand, the presence of high concentrations of TCEP in solution might introduce experimental artifacts. It was suggested in a few previous cases that strong reducing agents such as DTT and TCEP might generate H_2_O_2_ by a chain reaction of oxidation-reduction (redox) cycling [31,32], which could affect the enzyme kinetic constants and then produce both false positives and false negatives [33]. This might pose a risk for high-throughput screens to identify novel drug compounds for patients with albinism. 

According to our data, Tyrp1tr is in native conformation and adopts a structure like that of the crystal structure. First, our protein demonstrated DHICA activity in the presence of MBTH. Second, protein helical conformation was measured by circular dichroism at 222 nm and we have shown that Tyrp1tr is melted as a typical globular protein with the melting temperatures of 51 and 56 °C, at pH 7.2 and pH 5.5, respectively (Figure S6). Third, according to our sedimentation velocity data, the hydrodynamic diameter of 5.7 nm is expected for the protein with one layer of water, which is close to the value of 5.8 nm calculated from Tyrp1 atomic structure. In addition, we estimated the approximated frictional ratio of ~1.18, which is quite normal for a folded globular protein. An unfolded protein would have a much higher ratio [34].

However, our D_h_ values obtained in similar condition using DLS showed higher values of about 8 nm. Such discrepancies between DLS measurements and other methods are known [35]. In DLS, the hydrodynamic diameter defined as the diameter of a hypothetical hard-sphere that moves and diffuses in the same fashion as that of the protein macromolecule measured. The diameter is calculated from the diffusional properties of the protein, which has a complex non-spherical, dynamic (tumbling), and solvated shape. Therefore, the diameter is an apparent value different from a one determined from the protein structure. As a result, DLS for the samples incubated at 4 or 25°C will show a higher apparent value for hydrodynamic diameter. For this reason, we see that the D_h_ is larger compared to that obtained from sedimentation velocity, AFM, SAXS or from a direct calculation from the atomic model. Additionally, the DLS analysis based on the suggestion that the protein particle is spherical. The molecular surface area (MSA) of the Tyrp1 protein is 18300.4 Ȧ^2^. The diameter of the sphere with an “equivalent” surface area could be calculated from the equation, D = (MSA/π)^1/2^ producing the D of 7.63 nm. Therefore, with the addition of the glycans and the hydration shell to the protein atomic model, the D_h_ will be close to 8 nm, as it was measured from DLS. Similar results DLS has shown for other proteins of similar size [34,35].

In conclusion, we have studied the biochemical and biophysical properties of the Tyrp1 intra-melanosomal domain. We demonstrated that the domain is prone to aggregate at physiological temperatures. However, at the same time, the catalytic activity of Tyrp1tr raised significantly in a smaller volume of reaction decreased by the growth of protein aggregates. This effect could be important in the melanosome, especially at developmental stage IV when pigment production is intensified and the tyrosinases reaction volume will be decreased significantly due to melanin formation and protein aggregation. In the future, the mimicking of increased activity of tyrosinase could be a source for the creation of new “green” in vitro technologies to produce melanin and different components of the melanin pathway.

## 4. Materials and Methods

### 4.1. Tyrosinases Expression and Purification

The recombinant human intra-melanosomal domain of Tyrp1 (Tyrp1tr, residues 25–472) containing a HIS-tag on its C terminus, was expressed in baculovirus and commercially produced in whole insect *Trichoplusia ni* larvae (AllotropicTech, LLC, https://allotropictech.com/), then purified as previously described [5,14]. Briefly, after immobilized metal affinity (IMAC) and gel-filtration (GF) chromatography, Tyrp1tr was collected and concentrated using Amicon Ultra-15/10,000 NMWL centrifugal filter units (Millipore Sigma, Danvers, MA, USA) and then incubated with 1.5 M urea for 1 h at room temperature. Partially unfolded protein was applied to a Superdex 200 Increase GL 10/300 column (GE Healthcare, Pittsburg, PA, USA) and collected on an ÄKTApure liquid chromatography system equipped with UNICORN 7.0 software (GE Healthcare) as 0.5 mL fractions on a 96-well plate. The column was pre-calibrated with the GF standards (Bio-Rad, Hercules, CA, USA) thyroglobulin (670 kDa), γ-globulin (158 kDa), ovalbumin (44 kDa), myoglobin (17 kDa), and vitamin B12 (1.3 kDa). The fractions containing the peaks of interest were collected and concentrated using Amicon Ultra-15/10,000 NMWL centrifugal filter units. The protein concentration was determined using *A*260/280 nm measured with a NanoDrop 2000c UV–Vis spectrophotometer (Thermo Fisher Scientific, Waltham, MA, USA). Protein identity was confirmed by mass spectroscopy and Western blot analysis using anti-Tyrp1 antibodies (G17, Santa Cruz Biotechnology, Dallas, TX, USA).

### 4.2. Mass Spectroscopy

Pure Tyrp1tr was run on SDS-PAGE using 4–15% polyacrylamide gels (Bio-Rad) and stained using the Novex Colloidal Blue Stain Kit (Invitrogen, Thermo Fisher, Scientific). Liquid and in-gel digestions were performed on pre-alkylated protein bands using a method previously described [36] except for using acid extractable Sodium Dodecanoate [37]. Samples were off-line purified using Stage Tips [38]. Data collected by LC/MS/MS were analyzed using Mascot [39] against the extant NCBI protein data base without species specificity.

### 4.3. Dynamic Light Scattering

Dynamic light scattering (DLS) experiments were performed using the Litesizer 500 (Anton Paar USA, Ashburn, VA, USA). The protein at 2 mg/mL in GF buffer (50 mM Tris-HCl, pH 7.2 or 5.5, 1 mM EDTA, 150 mM NaCl, 50 µM TCEP) was measured in quartz cuvette QS 3.00 mm at the temperature range of 0–80 °C. For some experiments, proteins were pre-equilibrated in GF buffer at 4, 25, 31, 37, or 43 °C for 1 h or 24 h. The intensity curve fittings and the hydrodynamic diameter were performed and calculated using the Kalliope 2.2.3 software (Anton Paar Kalliope Professional, Ashburn, VA, USA).

### 4.4. Atomic Force Microscopy

Freshly cleaved mica disks were pre-treated with APS (amino-propyl-silatrane) as described previously [40]. Briefly, 12 mm mica disks were incubated with 0.17 mM APS (Aminopropyl-Silatrane) solution for 30 min, rinsed with 5 mL ultrapure water, and dried in a nitrogen gas stream. APS solution of treatment rendered the mica surface slightly hydrophobic and positively charged. Stock Tyrp1 samples were diluted to a protein concentration of ~100 nM and a 5 µL drop was deposited onto the APS treated mica and incubated for about 10 min before being lightly washed with ultrapure water (200–400 µL) and dried in a nitrogen stream. These samples were imaged on a MultiMode-8 AFM (Bruker-nano, Santa Barbara, CA, USA). Silicon probes (FESP_V2, Bruker-nano) with nominal stiffness of 2.8 N/m and resonance frequency of about 70 kHz were used for most of the imaging. Images were preprocessed on the Nanoscope Analysis v.1.9 software and then text images were exported to ImageJ (https://imagej.nih.gov/ij/) for particle analysis to identify and locate each protein particle. Each particle was then analyzed using Matlab code (Mathworks, Natick, MA, USA) to approximately deconvolve the probe size from the imaged particles to get closer to the real particle sizes. Origin software (OriginLab, Northampton, MA, USA) was used to analyze and present the statistics of the particle geometric parameters. Particle volume histograms were constructed, and the molecular mass of the particles was estimated assuming 35% *v/v* hydration water that remained bound in ambient air. The expected monomer volume of Tyrp1tr, assuming partial specific volume of 0.73 mL/gr and 35% *v/v* hydration in ambient air, was about 106 nm^3^.

### 4.5. Sedimentation Velocity

A Beckman Optima XL-I analytical ultracentrifuge, absorption optics, an An-60 Ti rotor, and standard double-sector centerpiece cells were used. Sedimentation velocity measurements of samples at 1 mg/mL, at 20 °C, were made at 40,000 rpm with data collection every 8 min to 3 h. Data analysis was done using DCDT+ 2.4.3. [41]. Correction of the sedimentation coefficient was made using protein partial specific volumes (ν-bar), calculated from the amino acid compositions, and solvent densities were estimated using the program SEDNTERP (http://www.rasmb.bbri.org/).

### 4.6. Tyrp1tr Enzymatic Assays

Tyrp1tr diphenol oxidase activity was measured spectrophotometrically using the SpectraMax i3 multi-mode detection platform (Molecular Devices, San Jose, CA, USA) and analyzed by SoftMax Pro software, rev. 6.5. The oxidation of DHICA to IQCA was measured at pH 7.2 or 5.5 using 3 mM DHICA (Santa Cruz Biotechnology) as a substrate in the presence of 3 mM MBTH [42,43]. The mixture was incubated for 180 min at 37 °C and then monitored at 505 nm.

### 4.7. Circular Dichroism

Thermal denaturation studies were done using a Jasco-715 spectropolarimeter equipped with a PTC-343W1 Peltier-type thermostatic cell holder. Circular dichroism was monitored at 222 nm using a 1-cm path-length cell with a Teflon stopper (Hellma). Cooling circulating water was supplied using a Neslab RTE-100 thermostatic circulator. Proteins (~ 0.1 OD 280 nm, in 10 mM sodium phosphate buffer, pH 7.2 and 5.5) were heated at 1–2 °C per minute with a temperature slope of 20–90 °C. The step resolution was 1 °C, the response time 1 sec, the bandwidth 2 nm and the sensitivity 100 mdeg. Temperatures at the transition midpoints, i.e., the melting temperature (Tm), were estimated from first derivative plots of the melting curves.

### 4.8. In-Silico DHICA Binding

The Tyrp1 atomic model, Tyrp1.pdb, was downloaded from the ocular proteomics website (https://neicommons.nei.nih.gov/#/proteome). The model was used to elucidate the mechanism of human TYRP1 and DHICA binding. The atomic model was subjected to 3 nanoseconds of molecular dynamics in YASARA (www.yasara.org), with conditions pH = 7.2, 0.9% NaCl, and a variable water density such that the pressure of the environment remained at 1 bar at the 5 different temperatures: 4, 25, 31, 37, and 43 °C. After molecular dynamics, the different PDB files were subjected to YASARA’s molecular docking script dock_run.mcr. The YASARA script was modified so that 200 docking runs were analyzed for each receptor–ligand pair instead of 25. The script uses AutoDock Vina, a gradient-optimization method holding the receptor (i.e., tyrosinase) rigid and the ligand (i.e., L-DOPA) flexible (Ref). It uses a statistical scoring function to give each binding conformation (of receptor and ligand), a binding affinity, and binding energy. The binding affinities were used for determination association constants at 5 different temperatures.

## Figures and Tables

**Table 1:** Tyrp1tr parameters determined from the gel-filtration chromatography and dynamic light scattering.

**pH 7.2**						
**Temperature (°C)**	**GF**				**DLS**	
	**Molecular Weight (kDa)**		**Area of Peak (%)**		**Hydrodynamic Diameter (nm)**	
	**Peak A**	**Peak B**	**Peak A**	**Peak B**	**Peak A**	**Peak B**
4	57.6 ± 1.9	-	98.2 ± 0.2	-	7.8 ± 0.1	-
25	59.6 ± 1.0	-	96.4 ± 1.2	-	8.5 ± 0.1	-
31	58.6 ± 1.4	961.3 ± 155.8	87.3 ± 1.0	8.7 ± 0.4	9.8 ± 0.8	-
37	56.5 ± 1.4	1074.8 ± 253.4	36.9 ± 0.3	48.7 ± 0.3	13.8 ± 0.5	52.8 ± 0.4
43	55.0 ± 1.8	1469.3 ± 500.1	7.6 ± 0.2	74.3 ± 1.8	30.4 ± 4.5	71.5 ± 1.3
**pH 5.5**						
**Temperature (°C)**	**GF**				**DLS**	
	**Molecular weight (kDa)**		**Area of peak (%)**		**Hydrodynamic diameter (nm)**	
	**Peak A**	**Peak B**	**Peak A**	**Peak B**	**Peak A**	**Peak B**
4	54.3 ± 0.9	-	93.6 ± 0.5	-	7.9 ± 0.6	-
25	55.0 ± 0.9	-	95.1 ± 0.6	-	7.6 ± 1.5	-
31	54.0 ± 0.5	1011.7 ± 16.5	88.8 ± 8.1	8.0 ± 8.8	16.1 ± 0.9	-
37	56.9 ± 0.9	989.1 ± 48.3	71.1 ± 3.2	13.8 ± 10.6	19.0 ± 0.6	-
43	57.6 ± 1.9	1023.6 ± 33.3	18.7 ± 3.1	17.9 ± 15.8	-	-

To estimate the molecular weight of Tyrp1tr (kDa), Superdex 200 Increase 10/300 column was pre-calibrated with the GF standards (Bio-Rad): thyroglobulin (670 kDa), γ-globulin (158 kDa), ovalbumin (44 kDa), myoglobin (17 kDa), and vitamin B12 (1.3 kDa). The Area of peaks (%) was measured using the UNICORN 7.0 software (GE Healthcare Bio-Sciences, Pittsburgh, PA, USA). The hydrodynamic diameter was calculated from DLS using the Kalliope Professional 2.2.3 software (Anton Paar, Graz, Austria).

## Supplementary Materials

Supplementary Materials can be found at https://www.mdpi.com/1422-0067/21/1/331/s1.

## Abbreviations

OCA1	oculocutaneous albinism Type 1 genetic disorder
OCA3	oculocutaneous albinism Type 3 genetic disorder
Tyrtr	intra-melanosomal domain of human tyrosinase
Tyrp1tr	intra-melanosomal domain of human tyrosinase related protein 1
IMAC	immobilized metal affinity chromatography
GF	gel-filtration (size-exclusion) chromatography
SV	sedimentation velocity
DLS	dynamic light scattering
AFM	atomic force microscopy
L-DOPA	L-3,4-dihydroxyphenylalanine
DHI	5,6-dihydroxyindole; IQ, indole 5,6-quinone
DHICA	5,6-dihydroxyindole-2-carboxylic acid
IQCA	indole-5,6-quinone-2-carboxylic acid
PTU	1-Phenyl-2-thiourea
TCEP	tris(2-carboxyethyl) phosphine
CHO	carbohydrate
