# Supplementary material for: Protein Stability and Functional Characterization of Intra-Melanosomal Domain of Human Recombinant Tyrosinase-Related Protein 1
Source: Int J Mol Sci. 2020 Jan 3;21(1):331. doi: 10.3390/ijms21010331 (PMC6981619; doi:10.3390/ijms21010331)
Supplement: Supplementary file 1 [file ijms-21-00331-s001.pdf]

Int. J. Mol. Sci. **2020**, *21*, 331; doi:10.3390/ijms21010331 www.mdpi.com/journal/ijms

Article

Protein Stability and Functional Characterization of Intra-Melanosomal Domain of Human Recombinant Tyrosinase-related Protein 1

Monika B. Dolinska [1], Kenneth L. Young II [1], Claudia Kassouf [1], Emilios K. Dimitriadis [2], Paul T. Wingfield [3], and Yuri V. Sergeev [1,*]

Supporting Information

Table S1. Temperature condition for analysis of the thermostability of Tyrp1tr.

Temperature (°C)	Condition
4	1) Protein short-term storage<br>2) Possible cold-denaturation[1, 2]
25	Room temperature for most experiments
31	Essential temperature for OCA1B related temperature-sensitive mutants[3]
37	Human body temperature
43	Heat shock temperature

[1]Privalov 1990 [1]
[2]Shan et al., 2010 [2]
[3]Dolinska et al., 2014 [3]

Table S2. Hydrodynamic diameter of Tyrp1tr at different temperatures.

Temperature (°C)	Hydrodynamic diameter (nm)
0	8.7 ± 0.1
4	8.7 ± 0.1
10	8.5 ± 0.1
20	8.3 ± 0.2
25	8.4 ± 0.4
30	8.4 ± 0.3
31	8.3 ± 0.1
32	8.3 ± 0.1
33	8.6 ± 0.2
34	9.0 ± 0.2
35	10.6 ± 0.6
36	13.8 ± 0.9
37	16.8 ± 0.7
38	19.3 ± 0.6
39	21.1± 1.0
40	23.5 ± 0.7
41	28.2 ± 2.0
42	36.6 ± 3.3
43	43.4 ± 2.3
44	47.2 ± 3.9
45	53.3 ± 3.6
50	110.1 ± 33.6
60	4298 ± 2719
70	5128 ± 2733

Hydrodynamic diameter measurement of Tyrp1tr was ascertained using the Litesizer 500 (Anton Paar USA, VA) and further calculated by Kalliope 2.2.3 software (Anton Paar Kalliope Professional). The gray shadows highlight the temperatures at which all other experiments were done: 4, 25, 31, 37, and 43°C.

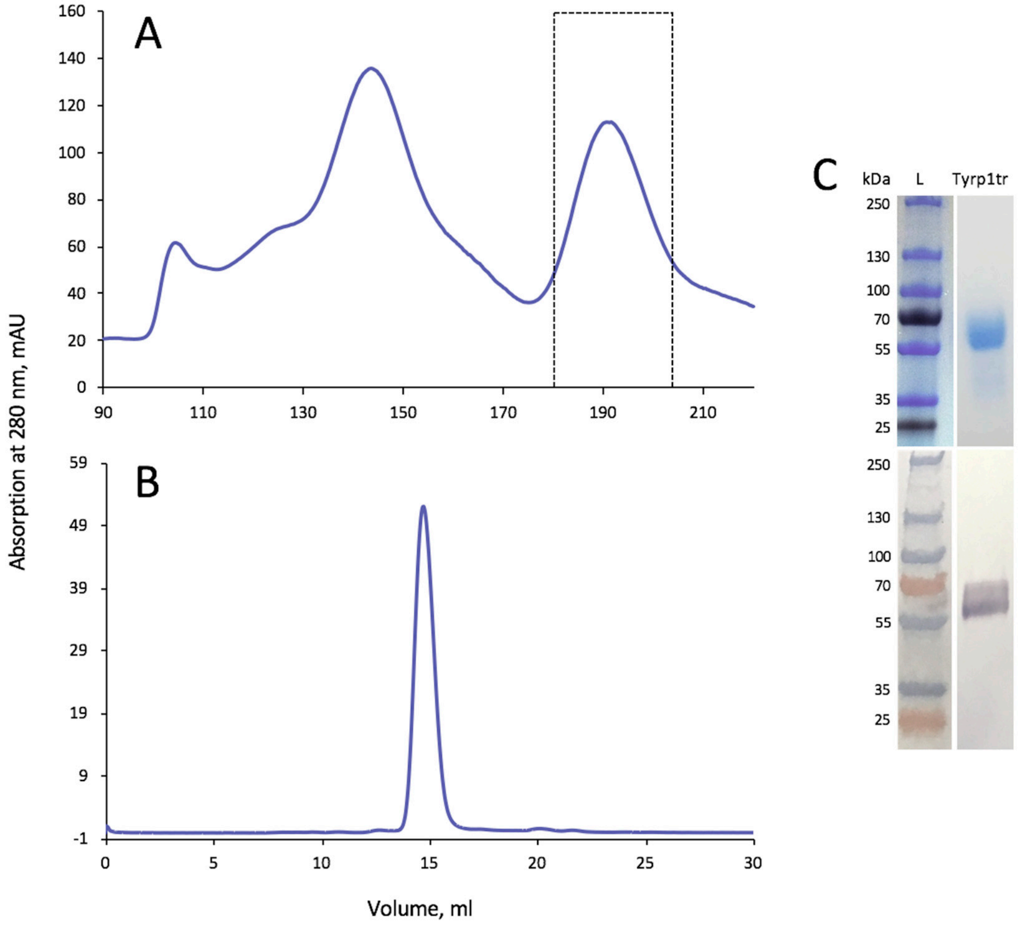

Figure S1. Purification of Tyrp1tr.
Panel A: Absorbance values at 280 nm recorded after two steps of purification using a ÄKTAxpress chromatography system. Tyrp1tr was eluted from a Sephacryl S-300 HR 16/60 column and its elution volume is shown by black dashed frame. **Panel B**: Pure Tyrp1tr eluted from a Superdex 200 Increase GL 10/300 column. **Panel C**: Pure Tyrp1tr SDS-PAGE (top panel) and Western blots performed using anti-Tyrp antibodies (G17, 1:500 dilution, Santa Cruz Biotechnology, CA). L stands for the protein ladder.

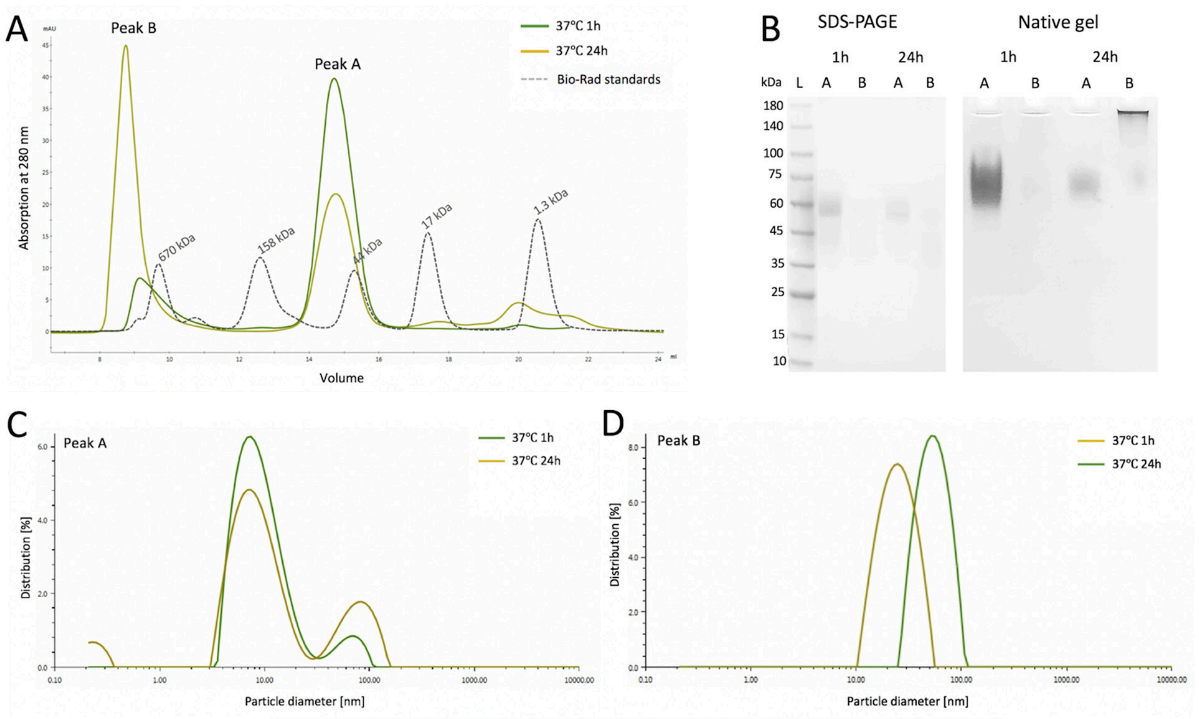

Time of incubation at 37°C (h)	GF				DLS	
	Molecular weight (kDa)		Area of peak (%)		Hydrodynamic diameter (nm)	
	Peak A	Peak B	Peak A	Peak B	Peak A	Peak B
1	58.6 ± 1.4	961.3 ± 155.8	81.6 ± 0.6	17.5 ± 0.6	8.7 ± 0.1	24.5 ± 0.7
24	56.5 ± 1.4	1074.8 ± 253.4	36.9 ± 0.3	48.7 ± 0.3	13.8 ± 0.5	52.8 ± 0.4

Figure S2. Time-dependent aggregation of Tyrp1 incubated at 37°C.
Panel A: GF performed with a Superdex 200 Increase 10/300 GL column for Tyrp1 incubated in Tris buffer, pH 7.4 (50 mM Tris-HCl, 150 mM NaCl, 1 mM EDTA, 50 µM TCEP) at 37°C for 1h (green line) and 24 hrs (yellow line). Tyrp1 either eluted as a monomer (Peak A) or higher molecular weight oligomers (Peak B). Grey line shows the Bio-Rad GF standards: thyroglobulin (670 kDa), γ-globulin (158 kDa), ovalbumin (44 kDa), myoglobin (17 kDa), and vitamin B-12 (1.3 kDa). **Panel B:** SDS-PAGE (left panel) and Native gel (right panel) of the concentrated fractions from Peak A (A) and Peak B (B) for the sample incubated at 37°C for 1 h or 24 hrs. **Panels C** and **D**: Dynamic light scattering of Tyrp1 incubated at 37°C for 1h (green line) and 24 hrs (yellow line) from peak A (**Panel C**) and peak B (**Panel D**). Table shows the molecular weight (kDa) and area of peaks (%) calculated from the gel filtration profile of Peak A and B eluted from a Superdex 200 Increase 10/300 GL column (GF) after incubation at 37°C for 1 and 24 hrs using the Unicorn 7.0 software (GE Healtcare, NJ). The hydrodynamic diameter (nm), polydispersity index (%), and diffusion coefficient (µm^2/s) of Peak A and B of Tyrp1 incubated at 37°C for 1 and 24 hrs were measured by dynamic light scattering (DLS) using Antoon Paar Kalliope Professional, version 2.2.3.

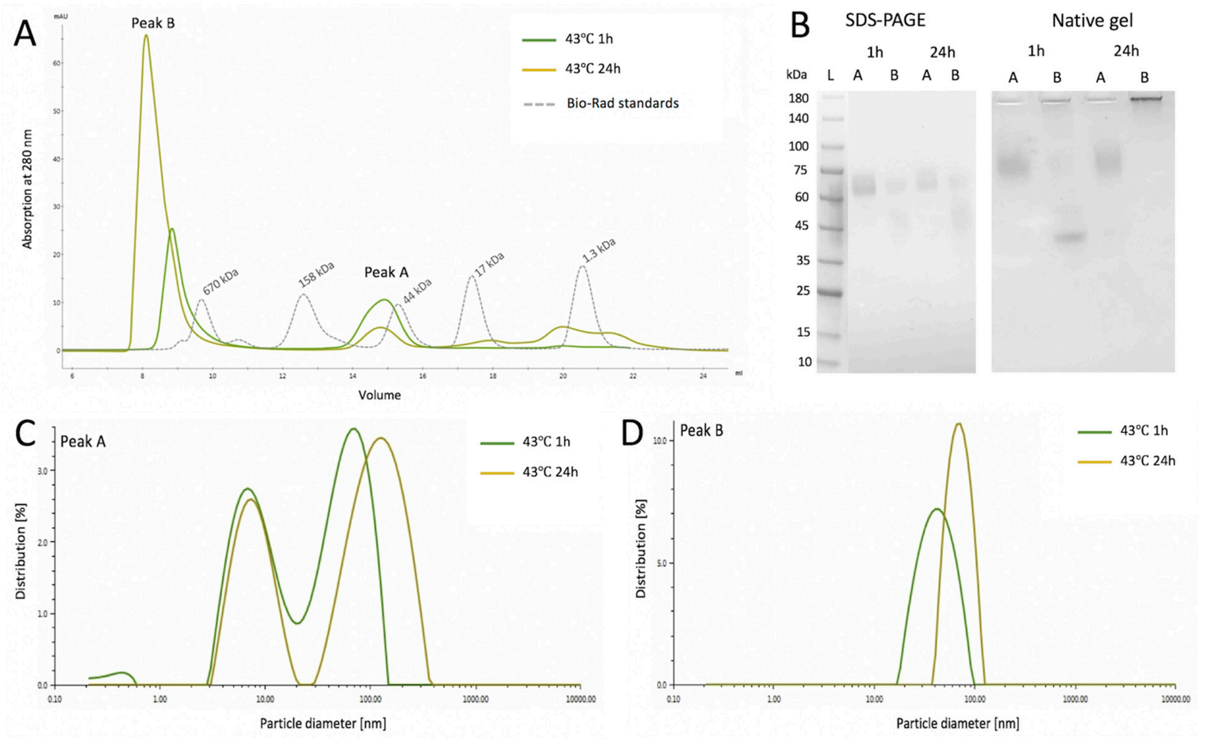

Time of incubation at 43°C (h)	GF				DLS	
	Molecular weight (kDa)		Area of peak (%)		Hydrodynamic diameter (nm)	
	Peak A	Peak B	Peak A	Peak B	Peak A	Peak B
1	51.9 ± 2.5	1144.1 ± 203.8	39.0 ± 0.3	58.9 ± 0.4	55.5 ± 16.4	40.0 ± 0.6
24	55.0 ± 1.8	1469.3 ± 500.1	7.6 ± 0.2	74.3 ± 1.8	30.4 ± 4.5	71.5 ± 1.3

Figure S3. Time-dependent aggregation of Tyrp1 incubated at 43°C.
Panel A: GF performed with a Superdex 200 Increase 10/300 GL column for Tyrp1 incubated in Tris buffer, pH 7.4 (50 mM Tris-HCl, 150 mM NaCl, 1 mM EDTA, 50 µM TCEP) at 43°C for 1h (green line) and 24 hrs (yellow line). Tyrp1 eluted either as a monomer (Peak A) or higher molecular weight oligomers (Peak B). Grey line shows the Bio-Rad GF standards: thyroglobulin (670 kDa), γ-globulin (158 kDa), ovalbumin (44 kDa), myoglobin (17 kDa), and vitamin B-12 (1.3 kDa). **Panel B:** SDS-PAGE (left panel) and Native gel (right panel) of the concentrated fractions from Peak A (A) and Peak B (B) for the sample incubated at 43°C for 1 h or 24 hrs. **Panels C** and **D**: Dynamic light scattering of Tyrp1 incubated at 43°C for 1h (green line) and 24 hrs (yellow line) from peak A (**Panel C**) and peak B (**Panel D**). Table shows the molecular weight (kDa) and area of peaks (%) calculated from the gel filtration profile of Peak A and B eluted from a Superdex 200 Increase 10/300 GL column (GF) after incubation at 43°C for 1 and 24 hrs using the Unicorn 7.0 software (GE Healtcare, NJ). The hydrodynamic diameter (nm), polydispersity index (%), and diffusion coefficient ($\mu m^2/s$) of Peak A and B of Tyrp1 incubated at 43°C for 1 and 24 hrs were measured by dynamic light scattering (DLS) using Antoon Paar Kalliope Professional, version 2.2.3.

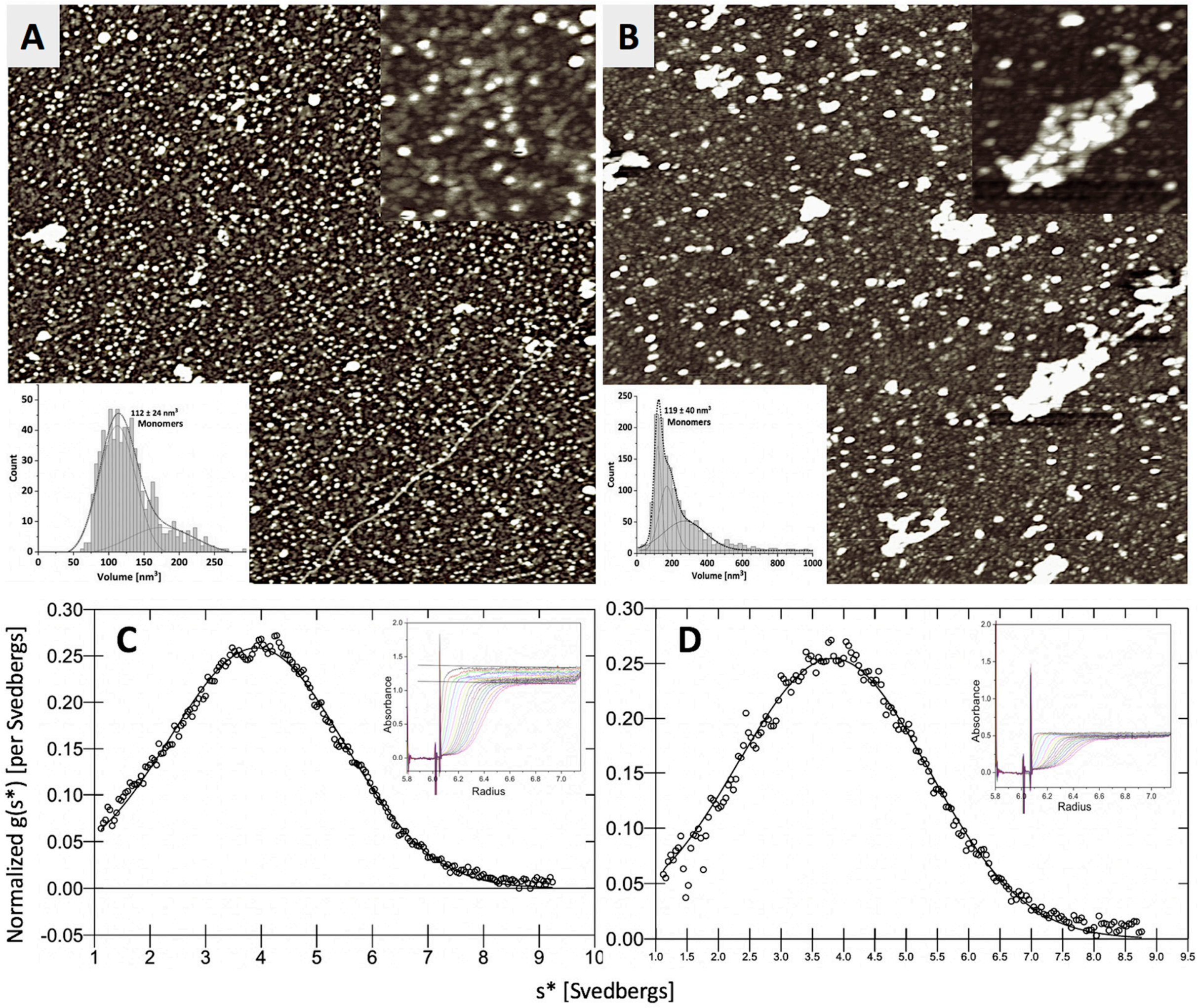

Figure S4. Atomic force microscopy and sedimentation velocity analysis of Tyrp1tr at pH 5.5.

Atomic force microscopy the full field images of the Tyrp1tr stored at 4ºC (**Panel A**) and pre-incubated at 37ºC for 24 h (**Panel B**). The inserted graph shows the particle volume distributions for the monomers (**Panel A**) The inserted pictures show the enlarged fragments of each image. Graphs below shows the sedimentation coefficient distributions in the samples stored at 4°C (**Panel C**) and pre-incubated at 37°C for 24 h (**Panel D**). Raw data are shown by open circles, a line represents the best fit. The inserts show the SV analysis of experiments conducted at 40,000 rpm, 20°C.

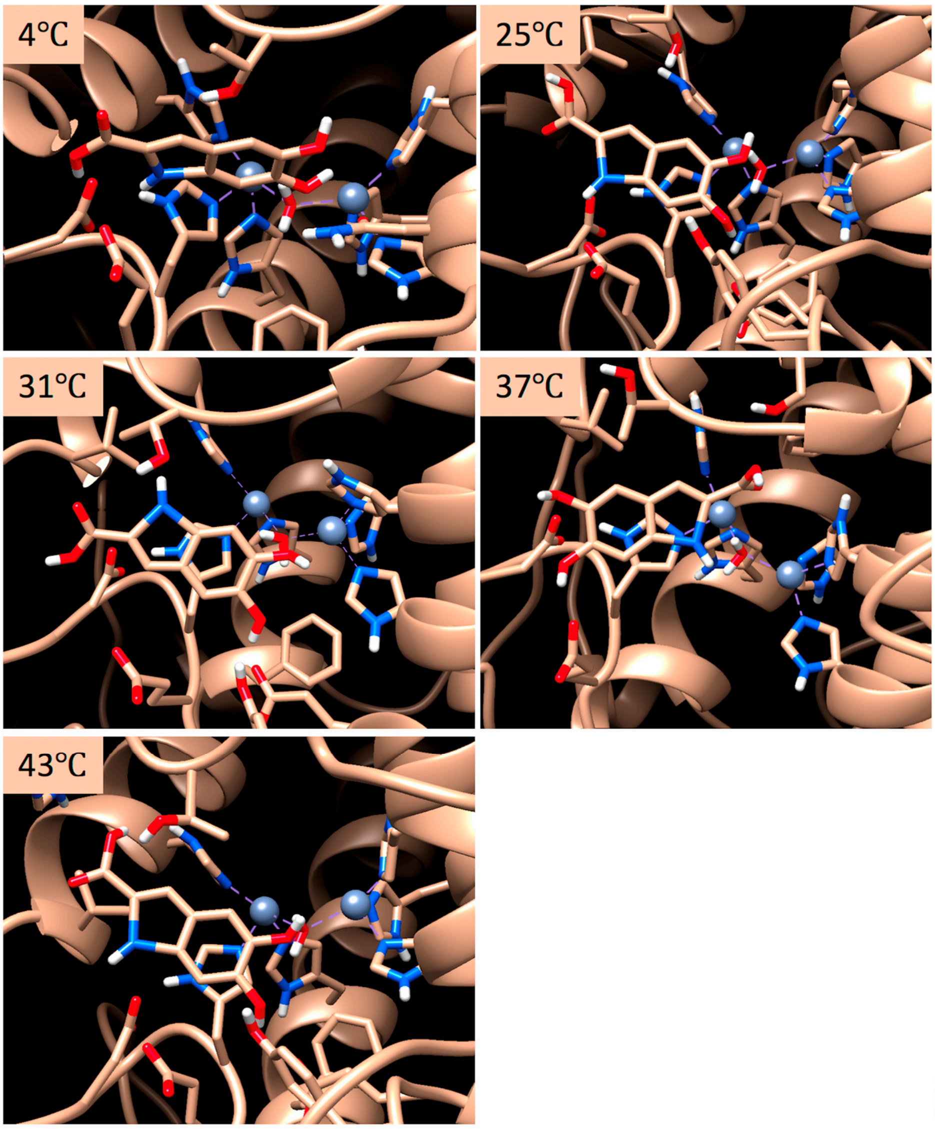

Figure S5. Computational docking of DHICA molecule and human Tyrp1.

The active centers of human Tyrp1 with DHICA docked at 4, 25, 31, 37, and 43°C are shown. Zn-atoms are shown as the blue spheres.

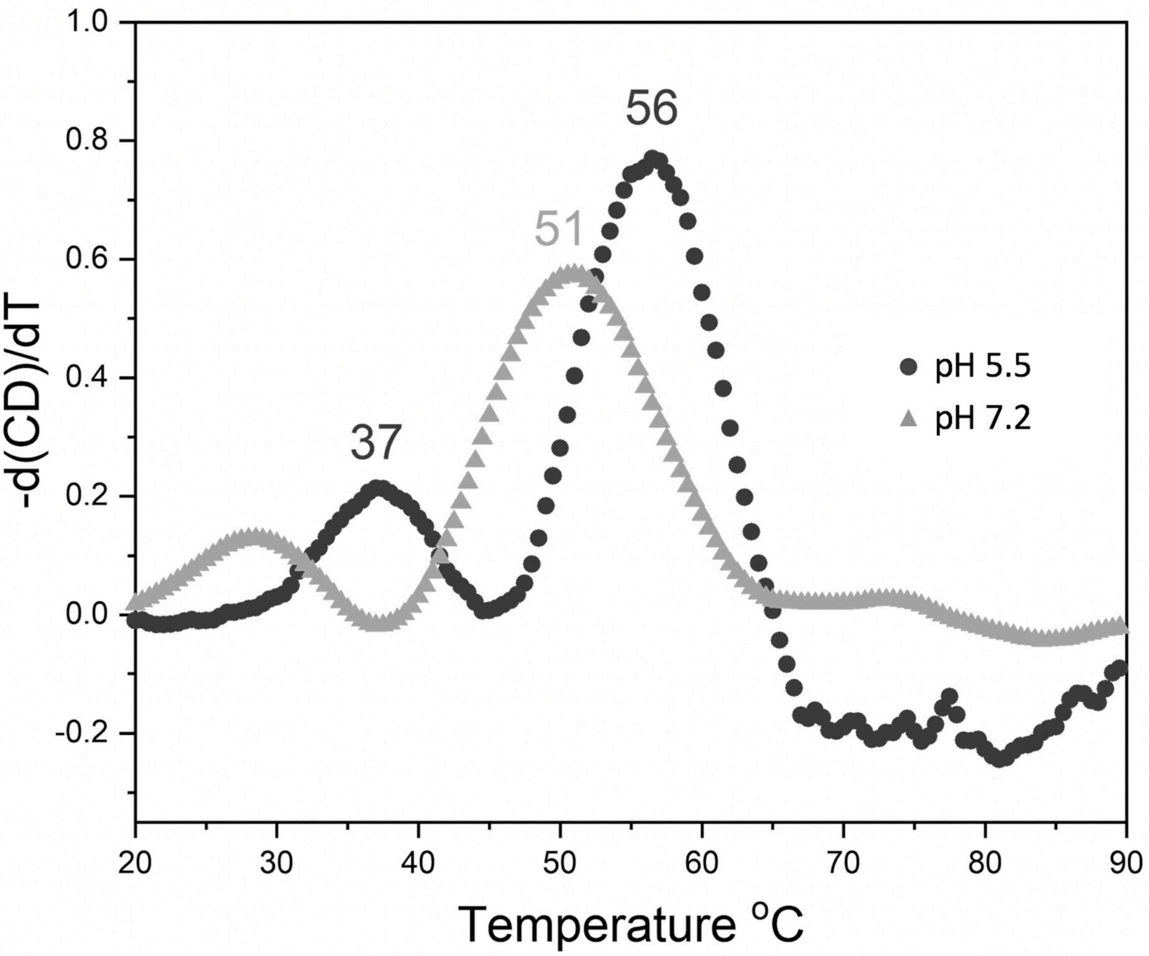

Figure S6. Thermal denaturation of Tyrp1tr at pH 5.5 and 7.2.

Tyrp1tr (~0.1 mg/ml) at pH 5.5 (black dots) and pH 7.2 (gray triangles) were scanned at 222 nm using a 1 cm path-length cell over the temperature range indicated. The midpoints of the transitions are the melting temperatures (Tm) and were estimated from first derivative of the curves indicated. Units of the ordinate are mean residue ellipticity [θ]mwr and have the dimensions: deg × dmol−1 × cm−2.

References

1. Privalov, P.L., *Cold denaturation of proteins.* Crit Rev Biochem Mol Biol, 1990. **25**(4): p. 281-305.
2. Shan, B., et al., *The cold denatured state of the C-terminal domain of protein L9 is compact and contains both native and non-native structure.* J Am Chem Soc, 2010. **132**(13): p. 4669-77.
3. Dolinska, M.B., et al., *Albinism-causing mutations in recombinant human tyrosinase alter intrinsic enzymatic activity.* PloS one, 2014. **9**(1): p. e84494.
